# Associations between psychoactive substance use and sensation seeking behavior among drivers in Norway

**DOI:** 10.1186/s12889-019-8087-0

**Published:** 2020-01-08

**Authors:** Ragnhild E. G. Jamt, Hallvard Gjerde, Håvard Furuhaugen, Giovanni Romeo, Vigdis Vindenes, Jan G. Ramaekers, Stig T. Bogstrand

**Affiliations:** 1 Department of Forensic Sciences, Oslo University Hospital, P.O. Box 4950, Nydalen, N-0424 Oslo, Norway; 2 Department of Nursing Science, Institute of Health and Society, University of Oslo, Oslo, Norway; 3 Department of Biostatistics, Oslo Centre for Biostatistics and Epidemiology, University of Oslo, Oslo, Norway; 4 Institute of Clinical Medicine, University of Oslo, Oslo, Norway; 5 Department of Neuropsychology and Psychopharmacology, Faculty of Psychology and Neuroscience, University of Maastricht, Maastricht, The Netherlands

**Keywords:** Driving under the influence (DUI), Sensation seeking, Psychoactive substances, Accidents

## Abstract

**Background/aim:**

Drug use and risky driving is associated with sensation seeking. The aim of this study was to investigate the association between use of psychoactive substances and levels of the sensation seeking personality trait as measured with the Brief Sensation Seeking Scale 4 among drivers in Norway.

**Method:**

A cross-sectional design was applied to estimate the association between psychoactive substance use and sensation seeking behavior. Drivers in normal traffic were included in two roadside surveys: one in the north (September 2014 – October 2015) and the other in the south-east of Norway (April 2016 – April 2017). Oral fluid was analyzed for alcohol and psychoactive drugs, and data on sex, age and time of participation were recorded. Participants filled in the Brief Sensation Seeking Scale 4 questionnaire.

**Results:**

A total of 8053 drivers were included, of which 32% were women and 62% were under 40 years. The prevalence of alcohol was 0.3%, stimulants 0.6%, tetrahydrocannabinol 1.4%, benzodiazepines and/or z-hypnotics 2.0% and polydrug use 0.6%. Associations were found between the use of tetrahydrocannabinol or benzodiazepines and/or z-hypnotics and a low score on the “thrill and adventure seeking” domain of the Brief Sensation Seeking Scale 4 (OR = 1.723, 95% C.I. = 1.001–2.966). Associations were also found between the use of stimulants and the highest scores on the “experience seeking” (OR = 2.085, 95% C.I. = 1.084–4.009) and “disinhibition” (OR = 4.791, 95% C.I. =1.748–13.135) domains of the Brief Sensation Seeking Scale 4. No associations were found between sensation seeking behavior and alcohol or polydrug use.

**Conclusion:**

A high degree of sensation seeking was found among drivers who had used stimulating drugs, in contrast to drives who had used tetrahydrocannabinol and benzodiazepines and/or z-hypnotics who showed a low degree of sensation seeking. The combination of sensation seeking behavior and the use of stimulants might lead to increased risky behavior and thus traffic crashes.

## Introduction

Throughout decades of research in the field of road traffic safety a number of different risk factors for traffic injury have been identified. Some of the main risk factors are speeding, driving under the influence (DUI) of alcohol, illicit or medicinal drugs, not wearing a seat-belt or helmet and demographic factors (sex and age) [1]. Previous Norwegian and Finnish studies on drivers arrested by the police for DUI found high degree of recidivism; over a period of 15 years (1984–1998) 71% of drugged drivers and 40% drunken drivers in a sample of arrested Norwegian drivers were rearrested one or more times [2]. In Finland, one third of drivers who had been arrested for suspected DUI of alcohol and/or drugs were rearrested within 15 years (1993–2007) [3]. Based on these and similar studies on recidivism among previously convicted DUI offenders it has been established that these drivers indeed are at higher risk of repeated DUI violations [4, 5]. As these drivers pose a serious threat of injury and death not only to themselves but also to other road users, research have been directed towards strategies for preventing both potential new cases and recidivists to commit DUI offences [6, 7].

In pursuit of uncovering what causes road traffic crashes, different kinds of analytical frameworks have been utilized to identify and analyze risk factors that are associated with road traffic crashes. One such framework commonly used in the field of road traffic safety is the Haddon matrix that identifies factors in different phases (pre-crash, crash, post-crash) and components (human, vehicle and equipment, environment) that determine the severity of injury in a road traffic crash [8].

Human factors have been identified to play a major role in causation of road traffic crashes in terms of the driver’s behavior. It has been estimated that driver-related behavioral factors (e.g. inexperience, drowsiness, alcohol and drugs use, psychological stress, speeding and overestimating capabilities) are the dominating cause in three out of five crashes and furthermore are contributing factors in the majority of other crashes [9, 10]. Because human factors have proven to be of great importance in targeting opportunities for harm reduction in the traffic, the driver’s behavior has been emphasized in traffic safety research.

Since the late 1940’s the driver’s personality has been a subject of interest in exploring what causes a driver’s behavior [11]. A personality trait that has been studied in relation to risky driving behavior is “sensation seeking”. This personality trait is defined by Zuckerman as “the seeking of varied, novel, complex, and intense sensations and experiences, and the willingness to take physical, social, legal, and financial risks for the sake of such experiences” [12]. Behaviors associated with this trait include engaging in unusual and risky acts, exceptional dangerous sports, fast and reckless driving, and use of alcohol and drugs. In addition to personality, it have been theorized that concepts such as attitude, social norms and perceived behavioral control play a central role in the prediction of behavior as described Ajzen in the theory of planned behavior (TPB) [13]. This theory has shown validity in explaining several behaviors related driving, such as drinking and driving [14–16]. Hence, willingness to drive under the influence of psychoactive substances may possibly be determined by an individual’s personality, perceived behavioral control, subjective norms and attitudes.

Because DUI offenders seem to be less susceptible to e.g. threats of sanctioning in terms of imprisonment, suspension of driver’s license, fines and/or educational programs than other drivers, it has been suggested that more tailored interventions based on a clearer understanding of underlying mechanisms, such as personality traits, that governs their behavior would be beneficial [7].

Sensation seeking is a personality trait that when measured can be a useful indicator of vulnerability to substance use disorders and propensity to engage in high-risk behavior such as risky driving [11, 17, 18]. In the present study we investigate the association between use of psychoactive substances by analysis of oral fluid, and levels of the sensation seeking personality trait among random drivers in normal traffic in Norway. We hypothesize that high and low levels of sensation seeking behavior are associated with different classes of psychoactive substances.

## Materials and methods

### Study design and setting

A cross-sectional design was applied to estimate the association between psychoactive substance use and sensation seeking behavior among Norwegian drivers. The drivers were recruited in two roadside surveys in cooperation with the local police and the Norwegian Mobile Police Service of Norway. The first of the two surveys was conducted in the arctic, rural county of Finnmark, the northernmost county of Norway, in the period from September 2014 to October 2015. The second of the surveys was conducted in five counties with both urban and rural areas in south-eastern Norway including Oslo, Akershus, Buskerud, Hedmark and Oppland from April 2016 to April 2017. In both surveys, drivers of cars, vans, motorcycles and mopeds were included. The six above mentioned counties have a total of approximately 2.0 million inhabitants, which accounts for about 38% of the Norwegian population.

### Participants

Because rural areas in Norway are sparsely populated, roadside sampling could not be done completely random in neither of the two surveys. Hence, a three-stage cluster sampling procedure was used in both surveys.

The first stage of the sampling was to select major roads in the studied counties. For the survey in Finnmark, main roads with an annual average daily traffic of 500 motor vehicles or more were selected to achieve the desired number of participants. For the survey in south-eastern Norway, roadways included in the previous roadside survey performed in the same area in 2008–2009 formed the foundation when choosing roads for data collection [19, 20]. In the second stage, time intervals were systematically selected to cover all time periods of the week, and the third stage consisted of randomly stopping drivers in cooperation with the police to ask for participation. Further details on the sampling procedure of the roadside survey in Finnmark have been described by Jamt et al. [21] and of the roadside survey in south-eastern Norway by Furuhaugen et al. [19].

Participation in both surveys was voluntary and anonymous. Oral and written information was given to each driver. Informed consent was obtained verbally by all participants. A representative from the research project signed the consent with his or her own name on behalf of the participant to protect the anonymity of the participant. All drivers 18 years of age and older were eligible to participate, but professional drivers were not included in neither of the surveys. Both roadside surveys were approved by the Regional Committees for Medical and Health Research Ethics in Norway.

Based on numbers of participants in previous, comparable Norwegian roadside surveys and official statistics on traffic volume obtained from the Norwegian Public Road Administration it was estimated that 3000–5000 participants were needed to reach adequate statistical power [19, 21].

### Data sources/measurements

Whenever an informed consent was given, an oral fluid sample was collected for the analysis of psychoactive substances and a questionnaire was filled in. The questionnaire contained questions about the persons’ sex, age, citizenship, type of vehicle, geographical area, time of participation (2-h intervals), day of the week and month were also recorded. In addition, the drivers were asked to answer to what extent they agreed to four statements about sensation seeking (Brief Sensation Seeking Scale 4 (BSSS-4)) (Table 1) on a scale ranging from 0 (strongly disagree) to 4 (strongly agree). No personally identifiable data was collected.

**Table 1:** BSSS-4 items^a^

Domaine	Statement
1: Experience Seeking	I like to explore strange places on my own, even if it means getting lost
2: Thrill and Adventure Seeking	I sometimes like to do things that are a little frightening
3: Disinhibition	I like to have new and exciting experiences, even if they are unusual or illegal
4:Boredom Susceptibility	I prefer friends who are exciting and unpredictable

^a^ The wording of statements 1–3 are not exactly as those used by Stephenson et al. [22], but more in line with those originally used by Zuckerman et al. for the sensation seeking questionnaire (SSS-V) [23] in order to fit the translation into Norwegian better

Stephenson et al. has noted the potential drawbacks associated with the use of short measures such as the BSSS-4, as sensation seeking is apparent in a variety of behaviors and preferences [22]. More extensive measurements are advantageous with respect to the content validity and measurement errors. However, long measurements are not always the best suited option as in large-scale surveys. Stephenson et al. found that the BSSS-4 performed similarly to the more established measures of impulsive sensation seeking from the Zuckerman-Kuhlman Personality Questionnaire (*r*^*2*^ = 0.81) and the eight-item brief sensation seeking scale (*r*^*2*^ = 0.89) [22]. In the present study, all data was collected at the roadside which is a challenging place to gather data with respect to lengthy questionnaires. Because available space and time is particularly limited when collecting data at the roadside, we chose to use the BSSS-4.

### Collection and analysis of oral fluid

Samples of oral fluid were collected using Quantisal™ Oral Fluid Collection Device (Immunalysis Corporation, Pomona, CA) [19, 21]. The collection pad was placed in the mouth beside the cheek or under the tongue until the indicator turned blue (when absorption reached 1 ml of oral fluid) or until 5 min had passed. The samples were analyzed for alcohol with an automated enzymatic method [24], and for medicinal- and illicit drugs using ultra-high-performance liquid chromatography with tandem mass spectrometry detection as previously described [19, 21].

### Statistical testing

Comparison of proportions of the sexes**,** age groups (under/over the age of 40), time of day for participation (daytime = 8:00 a.m. – 7:59 p.m., nighttime = 8:00 p.m. – 7:59 a.m.), if participation took place during a weekday or weekend (weekday = Monday 8:00 a.m. – Friday 7:59 p.m., weekend = Friday 8:00 p.m. – Monday 7:59 a.m.) and prevalence of psychoactive substances between the two roadside surveys was performed using contingency tables with chi-square test. An average BSSS-4 sum score for each participant was calculated by summing the scores on each statement and dividing it by 4. These scores were classified as: low = average sum score ≤ 1, medium = 1 < average sum score < 3, high = average sum score ≥ 3. To test for differences in the BSSS-distribution between the surveys the proportions of average sum scores in each level were compared using a contingency table with chi-square test.

Adjusted odds ratios (OR) were calculated using multivariable logistic regression. Five different models were estimated where the dependent variable was either alcohol, tetrahydrocannabinol (THC), benzodiazepines and/or z-hypnotics, stimulants or polydrug use (yes/no, with “yes” corresponding to concentrations equal to or above the analytical cut-of limits). Covariates were sex (with “male” as reference), age (in completed years, with 18 years as reference), geographical area (with the roadsidesurvey in Finnmark as reference) and the four items of the BSSS-4. The items were included as ordinal categorical variables with three levels. The originally five-level scales were collapsed down to three levels as follows: the scores “1” (disagree), “2” (neutral) and “3” (agree) were collapsed into one level (reported as “medium” in Table 2) and set as reference, score “0” (strongly disagree) corresponding to level 1 (reported as “low” in Table 2) and “4” (strongly agree) corresponding to level 2 (reported as “high” in Table 2). This resulted in a three-level-scale consisting of the levels “strongly disagree”, “neutral” and “strongly agree”. The rationale for this classification was to separate those scoring the extreme values in both directions from the rest. Another reason for this classification was to gain statistical power and to simplify the interpretation of the results from the multivariable logistic regression analysis [25, 26].

**Table 2:** Characteristics of study participants

	Finnmark %	south-eastern Norway %	*P*	Total %
Sex				
Male	69.4	67.2	0.04	68.0
Female	30.6	32.8		32.0
Age groups				
< 40	40.0	37.2	0.012	61.8
≥ 40	60.0	62.8		38.2
Time of day				
Daytime (8:00 a.m. – 7:59 p.m.)	63.8	61.8	0.061	62.5
Nighttime (8:00 p.m. – 7:59 a.m.)	36.2	38.2		37.5
Period of the week				
Weekend	29.6	36.1	< 0.001	33.6
Weekday	70.4	63.9		66.4
BSSS-4 (average sum score)				
Low (< 1)	34.7	29.3	< 0.001	31.3
Medium (≥ 1 & ≤ 3)	60.6	64.8		63.2
High (> 3)	4.6	5.9		5.4

All statistical analyses were made in SPSS Statistics Version 23 (IBM Corporation, Armonk, NY). The level of statistical significance was *p* ≤ 0.05.

## Results

### Participants

Both roadside surveys included drivers of passenger cars, vans, motorcycles and mopeds. Of the invited drivers, 94% agreed to participate in Finnmark and 91% in south-eastern Norway. However, eight drivers were not illegible because of low age. Altogether, this resulted in a total of 8053 participating drivers; 5026 drivers from the roadside survey in south-eastern Norway and 3027 from the roadside survey in Finnmark [19, 21].

Data on either the sex of the driver, age, month, day of the week and time of the day for participation were missing for four of the participants. Data on the four sensation seeking items were missing or incomplete for 27 of the participants in the survey in Finnmark and 109 of the participants in the survey in south-eastern Norway. All the cases with incomplete data were included in the bivariate analysis where possible (Table 2) but excluded from the multivariable analysis.

There were small but statistically significant differences in gender and age distributions between the two surveys (Table 2); 69.4% of the participants in the survey performed in Finnmark were men versus 67.2% in the survey performed in south-eastern Norway (*p* = 0.04). 40.0% of the participants in Finnmark were under the age of 40 years versus 37.2% in the south-eastern Norway (*p* = 0.012). There were also significantly more samples collected during weekend in south-eastern Norway (36.1%) compared to Finnmark (29.6%) (*p* < 0.001). There were significant differences in the distributions of BSSS-4 average sum scores between the surveys; the proportion of average sum scores on the lowest level was higher in Finnmark than in south-eastern Norway. The proportions on the highest level were approximately the same in both surveys. Table 3 shows the substance groups that were analyzed in oral fluid samples. The prevalence of the substance groups among sexes and age groups (under/over the age of 40) in both surveys is also presented in Table 3. Significant differences were found in the prevalence of THC and polydrug use among female drivers between the two roadside surveys; significantly more female drivers were positive for THC and polydrug use in south-eastern Norway compared to Finnmark.

**Table 3:** Prevalence of alcohol and drugs in the roadside surveys in Finnmark and South-Eastern Norway

Substance	Prevalence Finnmark (%)	Prevalence south-east Norway (%)	*P*	Total
Alcohol (ethanol)	0.2	0.3	n.s.	0.3
Male	0.3	0.4	n.s.	0.4
Female	0	0.1	n.s.	0.04
< 40	0.2	0.3	n.s.	0.3
≥ 40	0.3	0.3	n.s.	0.3
Tetrahydrocannabinol (THC)	1.2	1.4	n.s.	1.4
Male	1.8	1.8	n.s.	1.8
Female	0.0	0.7	0.013	0.4
< 40	2.0	2.9	n.s.	2.5
≥ 40	0.7	0.6	n.s.	0.6
Stimulants	0.7	0.6	n.s.	0.6
Male	0.7	0.8	n.s.	0.8
Female	0.5	0.2	n.s.	0.3
< 40	0.9	1.2	n.s.	1.1
≥ 40	0.5	0.3	n.s.	0.3
Benzodiazepines and/or z-hypnotics	1.9	2.1	n.s.	2.0
Male	1.6	1.3	n.s.	1.4
Female	2.4	3.6	n.s.	3.2
< 40	0.7	0.6	n.s.	0.7
≥ 40	2.6	2.9	n.s.	2.8
Other	0.7	0.5	n.s.	0.6
Male	0.6	0.5	n.s.	0.5
Female	0.8	0.6	n.s.	0.6
< 40	0.2	0.2	n.s.	0.2
≥ 40	0.9	0.7	n.s.	0.7
Polydrug	0.6	0.6	n.s	0.6
Male	0.8	0.6	n.s	0.7
Female	0.1	0.7	0.046	0.5
< 40	0.6	0.7	n.s	0.7
≥ 40	0.6	0.5	n.s	0.5

### Estimation of associations between sensation seeking behavior and drug use among drivers

Adjusted odds ratios for finding alcohol, THC, benzodiazepines/z-hypnotics or stimulants in oral fluid were calculated by considering four multivariable logistic regression models for the different substance groups.

We first estimated full models including as covariates: sex, age, geographical area (north or south-east) and the four subscales of the BSSS-4 (each subscale included as a categorical variable with five levels). Then with a backward stepwise procedure reduced models were selected. This procedure found the effect of the subscales “experience seeking” and “boredom susceptibility” and the variable “geographical area”” not to be statistically significant in the model with use of THC as dependent variable (F-test = 14.4 and *p*-value = 0.11). In the model with use of stimulants as dependent variable there were no significant associations with the subscale “boredom susceptibility” and the variable “geographical area”” (F-test = 0.97 and *p*-value = 0.97). The model with use of benzodiazepines and/or z-hypnotics as dependent variable, the procedure found the effect of the subscale “disinhibition” and “geographical area” not to be statistically significant (F-test = 5.08 and *p*-value = 0.41). None of the variables except for the “sex” variable in the model with the use of alcohol as dependent variable were significant.

Furthermore, new reduced models with the BSSS-4 subscales classified into three levels, as described, were estimated (Table 4). From the new models, statistically significant associations were found between scoring the lowest level on the “thrill and adventure seeking” subscale and the use of THC, and benzodiazepines and/or z-hypnotics. Statistically significant associations were also found between scoring the highest level on the “experience seeking” and “disinhibition” subscales and the use of stimulants.

**Table 4:** Multivariable analysis of sensation seeking behavior and the use of THC (model 1), stimulants (model 2) and benzodiazepines and/or z-hypnotics (model 3) among drivers

Variables	Model 1 (THC)			Model 2 (Stimulants)			Model 3 (Benzodiazepines and/or z-hypnotics)		
	Adjusted OR	95% C.I.	*p*	Adjusted OR	95% C.I.	*p*	Adjusted OR	95% C.I.	*p*
Sex	0.22	0.11–0.41	< 0.01	n.s.	n.s.	n.s.	2.53	1.83–3.51	< 0.01
Age	0.95	0.93–0.96	< 0.01	0.96	0.93–0.98	< 0.01	1.06	1.05–1.07	< 0.01
Experience Seeking, reference									
Experience Seeking, level 0	n.s.	n.s.	n.s.	n.s.	n.s.	n.s.	n.s.	n.s.	n.s.
Experience Seeking, level 4	n.s.	n.s.	n.s.	2.09	1.08–4.01	0.03	n.s.	n.s.	n.s.
Thrill and adventure seeking, reference									
Thrill and adventure seeking, level 0	1.72	1.00^a^-2.97	0.05	n.s.	n.s.	n.s.	1.50	1.01–2.24	0.05
Thrill and adventure seeking, level 4	n.s.	n.s.	n.s.	n.s.	n.s.	n.s.	n.s.	n.s.	n.s.
Disinhibition, reference									
Disinhibition, level 0	n.s.	n.s.	n.s.	n.s.	n.s.	n.s.	–	–	–
Disinhibition, level 4	n.s.	n.s.	n.s.	4.79	1.75–13.14	< 0.01	–	–	–
Boredom Susceptibility, reference									
Boredom Susceptibility, level 0	–	–	–	–	–	–	n.s.	n.s.	n.s.
Boredom Susceptibility, level 4	–	–	–	–	–	–	n.s.	n.s.	n.s.

*n.s.* Not significant, − Not included in the reduced models, ^a^ rounded off from 1001

## Discussion

In this study the association between use psychoactive substances and levels of sensation seeking behavior among drivers in the northernmost part and the south-eastern part of Norway has been investigated using a cross-sectional approach.

Through logistic regression, we found statistically significant associations between the use of stimulants and scoring the highest level on the subscales “experience seeking” and “disinhibition” of the BSSS-4 adjusting for the effects of sex, age and geographical area. We also found that the use of THC, benzodiazepines and/or z-hypnotics was significantly associated with scoring the lowest level on the subscale “thrill and adventure seeking”.

These results are in line with findings in previous studies with respect to the relationship between sensation seeking and use of stimulants. On the subscale level, Carrol and Zuckerman found that stimulant use was significantly correlated with the “disinhibition” and “boredom susceptibility” subscales [27], and Huba et al. found significant associations between the “experience seeking” and “boredom susceptibility” subscales and the use of stimulants [28]. Harvanko et al. found that sensation seeking dimension of the Zuckerman-Kuhlman Personality Questionnaire was positively associated to the level of reinforcement produced by a single dose administration of amphetamine effects [29]. Sáiz et al. found that both experimental and frequent cocaine users had significantly higher sensation seeking scores compared to non-users in a sample of secondary school students [30]. However, Spotts and Shontz found in their study that amphetamine users had the highest sensation seeking scores compared to barbiturate users, nonusers and opiate users, while cocaine users had the lowest score [31].

As in the present study, the “thrill and adventure seeking” subscale has been found to be negatively related to the use of marijuana and sedatives [28]. However, the same subscale was found to be positively related to the use of cannabis (hasjish, marijuana). The authors have explained this seemingly contradictory relationship between the “thrill and adventure seeking” subscale and the use of cannabis and marijuana by differences in usage patterns; the group of more committed/chronic users of marijuana does not tend to seek out frightening or risky activities that would typically result in high scores on the “thrill and adventure seeking” subscale. The “experience seeking” and “disinhibition” subscales have been found to be positively related to the use of cannabis and psychosedative prescription drugs like barbiturates and benzodiazepines [28, 32]. In the present study no such associations were found.

The conflicting results between studies that have investigated the relationship between sensation seeking behavior and drug use/drug preference have been noted previously and several factors have been pointed out to influence the results like selection of subjects that were studied (e.g. young, nondependent users versus chronic users, different types of “uppers” and “downers” and poly-drug use versus single-drug use [33, 34]).

It is well known from the literature that alcohol and psychoactive drugs can impair the ability to operate motorized vehicles as a result of inhibited reaction time, tracking, vigilance, attention and visual function [35]. In addition, alcohol and psychoactive substances may influence on a driver’s emotional behavior such as judgment, aggression and risk taking. Previous studies have shown that individuals who exhibit sensation seeking behavior are more likely to drive under the influence of alcohol and drugs [36]. Sensation seeking personality trait is not only associated with the use of alcohol and drugs, which in its own right may induce risk taking behavior, it is also known to be related to different aberrant driving behaviors like speeding and driving more aggressively regardless of whether or not the driver is under the influence of psychoactive substances. Inherently this behavior alone poses a threat to traffic safety.

The results of the present study suggest that drivers who use psychoactive substances vary in their preference for sensation seeking. This result is in line with the findings of previous research that drivers prone to engage in risky driving behavior such as DUI of psychoactive substances, may vary in their preference for risk-taking [7]. It has also been found that these drivers vary in their susceptibility to preventive measures. Hence, they should not be looked upon as a homogenous group when countermeasures are considered.

### Limitations

An important strength of this study is that we could estimate the association between sensation seeking behavior and psychoactive substance by specific chromatographic analysis of oral fluid samples and not self-reported use of psychoactive substances. Another strength is that the participation rates in the roadside surveys were high, above 90%.

In our study we found no association between testing positive for alcohol use and sensation seeking behavior. A number of studies have demonstrated an association between alcohol impaired driving and sensation seeking behavior [11, 37, 38], hence it is reasonable to assume that such an association also may exist among Norwegian drivers. The lack of association is likely due to a low number of alcohol positive drivers in this study (*n* = 22) and the result might have turned out differently if our study sample had been larger. The same was the case for polydrug use; no association was found with sensation seeking behavior. Polydrug users have been found to be higher in sensation seeking than both non-users and depressants users [33, 39]. As with the alcohol positive drivers the number of drivers positive for more than one drug was rather low (*n* = 48). Because the participation in the roadside surveys was voluntary, it is likely that some drivers, who had recently used alcohol or drugs of abuse not revealed by the police, might have rejected to participate in fear of being prosecuted.

A well-known concern using questionnaires is the phenomenon of socially desirable response (SDR) bias; that is “the tendency for participants to present a favorable image of themselves” [40]. This may confound the results and lead to inaccurate conclusions.

As have been outlined above, lengthy questionnaires were practically impossible to conduct in the roadside surveys of the present study and so we have not been able to measure the degree of SDR bias in our results. Although the participation in the roadside surveys was anonymous, it might be that the context our data were collected in (i.e. the police were present) may have contributed to bias the results in the direction of lower scores on the sensation seeking.

## Conclusion

A high degree of sensation seeking was found among drivers who had used stimulating drugs, in contrast to drives who had used THC and benzodiazepines and/or z-hypnotics who showed a low degree of sensation seeking. The combination of sensation seeking behavior and the use of stimulants might lead to risky behavior and thus traffic crashes. This may have implication for traffic safety and deterrence measures such as specially developed educational programs that aims specifically at high-sensation seekers with regard to risky driving behavior, including driving under the influence of psychoactive substances.

## Data Availability

Data will not be shared due to an institutional agreement.

## Abbreviations

BSSS-4: Brief sensation seeking scale 4
CI: Confidence interval
DUI: Driving under the influence
OR: Odds ratio
SDR: Socially desirable response
THC: Tetrahydrocannabinol
